# The distribution of benthic amphipod crustaceans in Indonesian seas

**DOI:** 10.7717/peerj.12054

**Published:** 2021-08-30

**Authors:** Tri Arfianti, Mark John Costello

**Affiliations:** 1 Research Center for Biology, Indonesian Institute of Sciences, Cibinong, Jawa Barat, Indonesia; 2 School of Environment, University of Auckland, Auckland, New Zealand; 3 Faculty of Bioscience and Aquaculture, Nord University, Bodø, Norway

**Keywords:** Indonesia, Biodiversity, Biogeography, Taxonomy, Amphipoda, Distribution

## Abstract

Amphipod crustaceans are an essential component of tropical marine biodiversity. However, their distribution and biogeography have not been analysed in one of the world’s largest tropical countries nested in the Coral Triangle, Indonesia. We collected and identified amphipod crustaceans from eight sites in Indonesian waters and combined the results with data from 32 additional sites in the literature. We analysed the geographic distribution of 147 benthic amphipod crustaceans using cluster analysis and the ‘Bioregions Infomaps’ neural network method of biogeographic discrimination. We found five groups of benthic amphipod crustaceans which show relationships with sampling methods, depth, and substrata. Neural network biogeographic analysis indicated there was only one biogeographic region that matched with the global amphipod regions and marine biogeographic realms defined for all marine taxa. There was no support for Wallaces or other lines being marine biogeographic boundaries in the region. Species richness was lower than expected considering the region is within the Coral Triangle. We hypothesise that this low richness might be due to the intense fish predation which may have limited amphipod diversification. The results indicated that habitat rather than biogeography determines amphipod distribution in Indonesia. Therefore, future research needs to sample more habitats, and consider habitat in conservation planning.

## Introduction

Species distribution data are the foundation for understanding global patterns of biodiversity, prioritizing areas for conservation, and projecting the impacts of habitat loss and climate change (*e.g.*, Zhao et al., 2020). Amphipods are one of the most abundant and diverse macro-invertebrates in the oceans, playing a major role in food webs as herbivores, predators, detritivores, and scavengers, and as food for other species such as macroinvertebrates and fish (Arfianti & Costello, 2020a).

Indonesia lies within the Coral Triangle, an area considered to have the highest richness of corals, and the highest richness and endemicity of fish, in the world (Asaad et al., 2018; Lohman et al., 2011). Marine fishes, lobsters, corals, and other marine biota are reported to have the highest biodiversity in Indonesia (Allen, 2008; Carpenter & Springer, 2005; Hoeksema, 2007; Roberts et al., 2002; Veron et al., 2009). Thus, it is expected that Indonesia will have a high diversity of amphipods.

**Figure 1:**
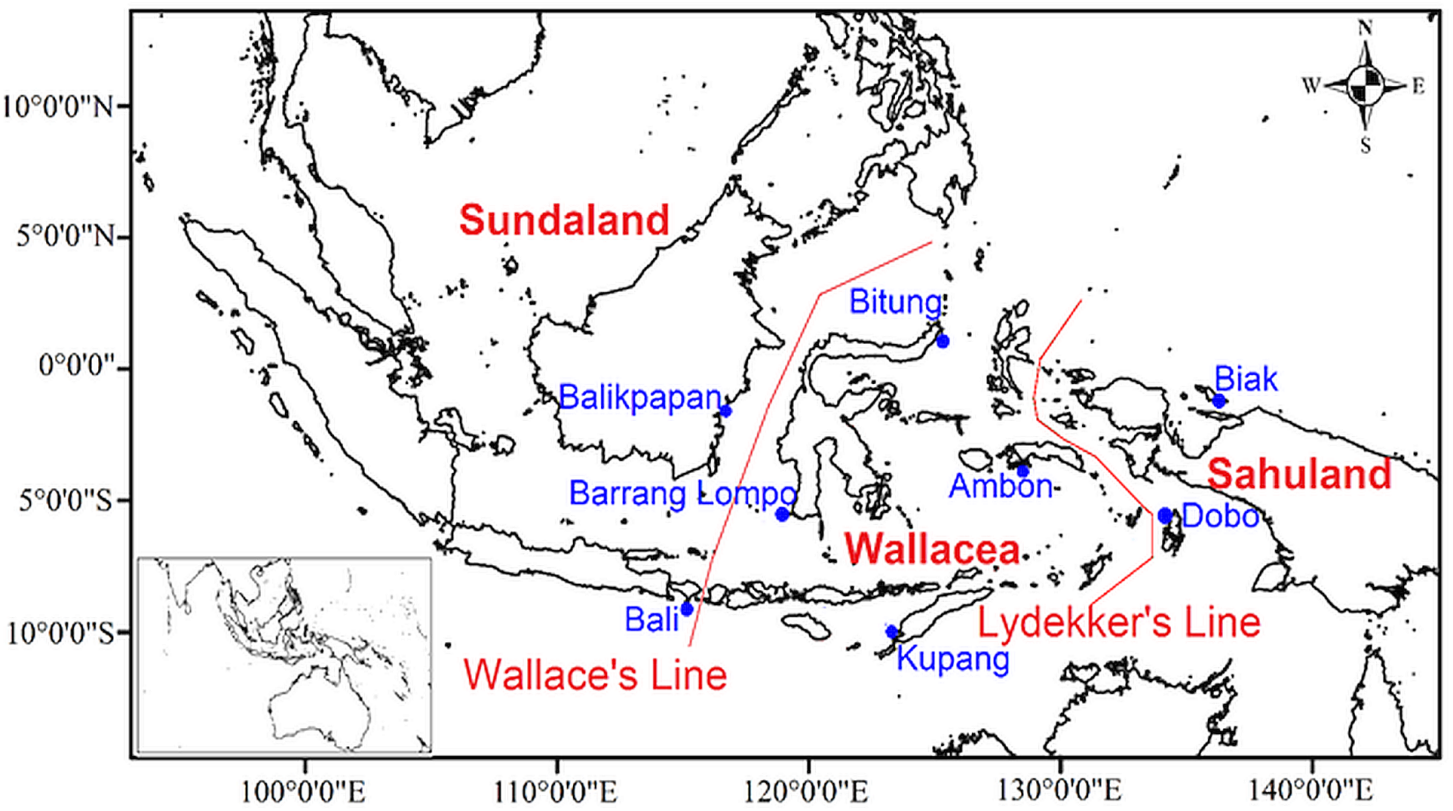
Map of sampling locations in eight sites of the Indonesian archipelago showing Wallace’s and Lydekker’s lines.

Several biogeographic boundaries were proposed within Indonesia. Based on Wallace’s hypothesis Wallace (1860), Dickerson (1928) named the Moluccas, Sulawesi, and the Lesser Sunda Islands as the Wallacea region (Fig. 1). Geologically, Wallacea is formed from fragments of Gondwana and Australian crust by numerous collisions since the late Palaeozoic (Hall & Sevastjanova, 2012). Collisions and plate tectonic movements influenced the bathymetry and topography changes, and land or sea distributions (Hall, 2009). These movements are expected to have drawn the Australian and Asian fauna and flora closer together in Wallacea, thereby enriching the biota (Hall, 2012; Michaux, 2010). Additional biogeographic boundaries, namely Huxley’s Line, Lydekker’s Line, and Weber’s Line, were proposed in Indonesia (Simpson, 1977). The islands of Wallacea have numerous endemic terrestrial faunas since they were not connected to any surrounding land (either Sundaland or Sahuland) during the lower sea levels in the Late Pleistocene and Holocene (Lohman et al., 2011; Sodhi et al., 2004). Thus, Wallacea is considered a natural laboratory of evolutionary processes and faunal diversification (Lohman et al., 2011; Voris, 2000).

Studies on terrestrial vertebrates supported Wallace’s Line as a biogeographic boundary for species dispersal (Oliver et al., 2015; Procheş & Ramdhani, 2012). However, most other studies on terrestrial species (Table S1) did not support Wallace’s hypothesis about the boundary line. Similarly, most studies on marine species generally did not support a marine Wallace’s Line (Table S1). Nevertheless, whether Wallace’s Line is a barrier for amphipods has never been studied. As amphipods have direct development and no planktonic larva, their dispersal is expected to be limited and may result in high rates of endemicity capacity (Marques & Bellan-Santini, 1990). In this paper newly collected and existing data on the distribution of these crustaceans in Indonesia were analysed. We use cluster and neural network analysis to map their biogeography and we compared the richness of species to other regions.

## Methods

### Field sampling

Amphipods were collected at eight locations in Indonesia as part of the present qualitative study, namely Ambon (Maluku Islands), Biak Island (Papua), Bitung (North Sulawesi), Barrang Lompo (Spermonde Archipelago), Bali, Kupang (East Nusa Tenggara), Balikpapan (East Kalimantan), and Dobo (Aru Islands) (Fig. 1). At each study site (Fig. 1), amphipods were sampled by hand while wading during the lowest tide at daytime from algae and coral rubble, placed in a bucket filled with seawater and clove oil, and covered with a lid. After one hour, all amphipods were unconscious and then sieved on a 0.5 mm sieve and transferred to a 250 ml wide-mouth bottle with 75% ethanol and filtered seawater.

**Figure 2:**
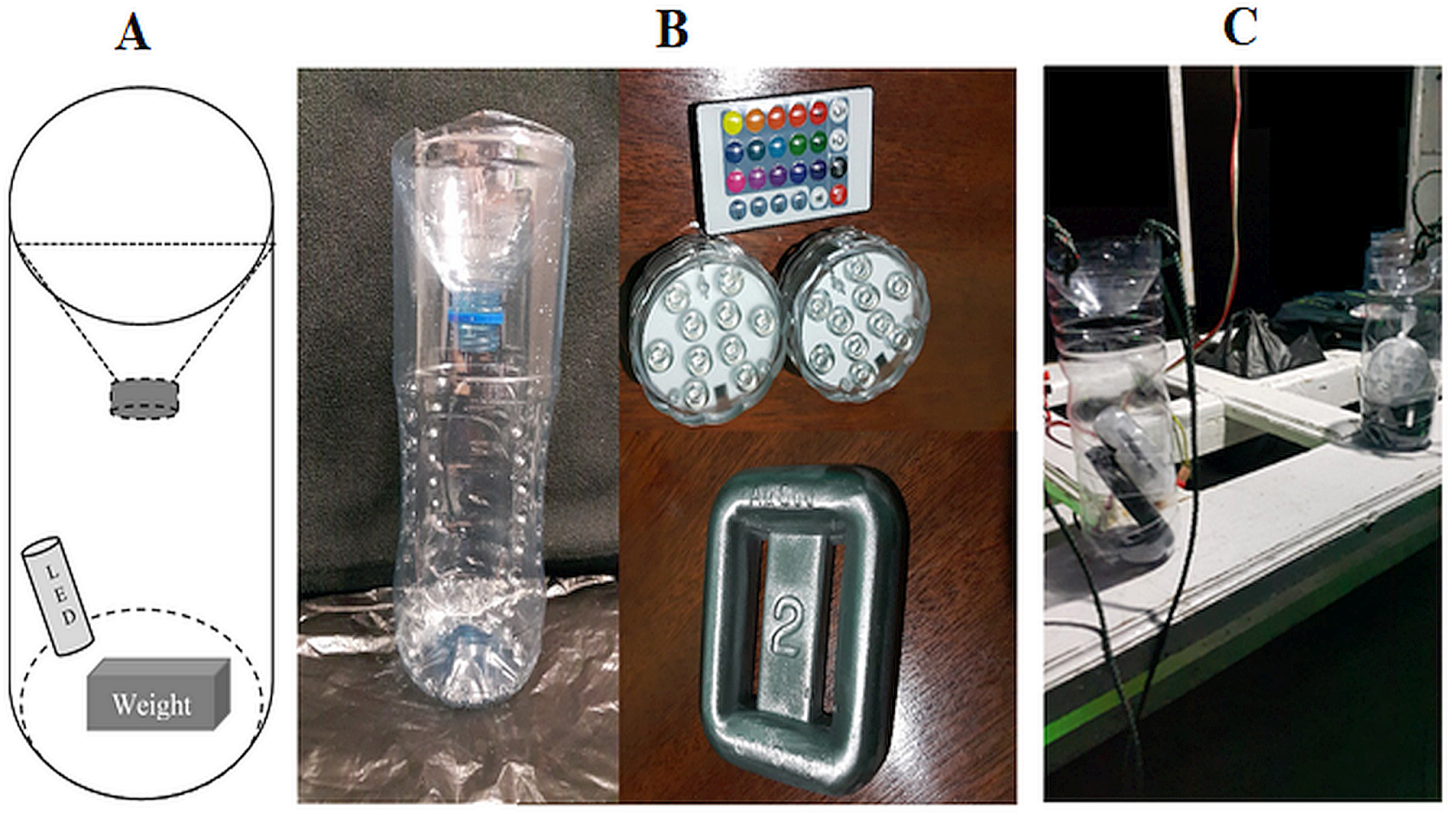
The light traps used in this study. (A) Illustration of the dimensions and structure, (B) Photograph of the components used to assemble the light traps *i.e.,* 1.5 L water bottles, remote controlled LED lights (top), a 2 kg lead weight (bottom), and (C) Light traps on a boat ready for deployment.

In addition to hand collection of samples, two sets of simple light traps using 1.5 L water bottles containing LED lights (Fig. 2) were placed at more than 10 m apart from each other. These traps were anchored to the subtidal seabed among coral reef substrata using a 2 kg lead weight during the lowest tide at night for at least one hour. All collections were conducted in locations which were remote from houses or pathways lights so that the presence of ambient-artificial light was eliminated. However, the phases of the moon varied on the sampling dates (Table 1). After sampling, large animals (*e.g.*, juvenile fish) were removed, and the remaining catch was retained on a 0.5 mm sieve. Any spilled or excess sample was caught in the bucket and was re-sieved. Specimens were transferred to a 250 ml wide-mouth bottle and preserved in 75% ethanol. Abundant catches of amphipods (>2,000 individuals) were sub-sampled in the laboratory. The bottle was gently rotated to evenly mix the specimens before removing a subsample using a pipette. Representative specimens were removed by hand and identified. Juvenile and damaged individuals were not included in the analysis. The identification of individuals was made to the lowest taxonomic level using relevant publications (Barnard, 1969; Barnard & Karaman, 1991a; Barnard & Karaman, 1991b; Bowman, 1973; Coleman, Lowry & Macfarlane, 2010; Laubitz, 1991; Lowry & Myers, 2009; Lowry & Myers, 2013; Lowry & Stoddart, 1993; Ortiz & Lalana, 1997; Ortiz & Lalana, 1999; Ortiz & Lalana, 2003; Pirlot, 1932; Pirlot, 1933; Pirlot, 1934; Pirlot, 1936; Pirlot, 1938; Ren, 2006; Ren, 2012; Vinogradov, Volkov & Semenova, 1996; Watling, 1989). However, some amphipods were missing diagnostic characters due breakage and thus only identifiable to genus level (Table 2). Nomenclature follows Horton et al. (2021).

**Figure 3:**
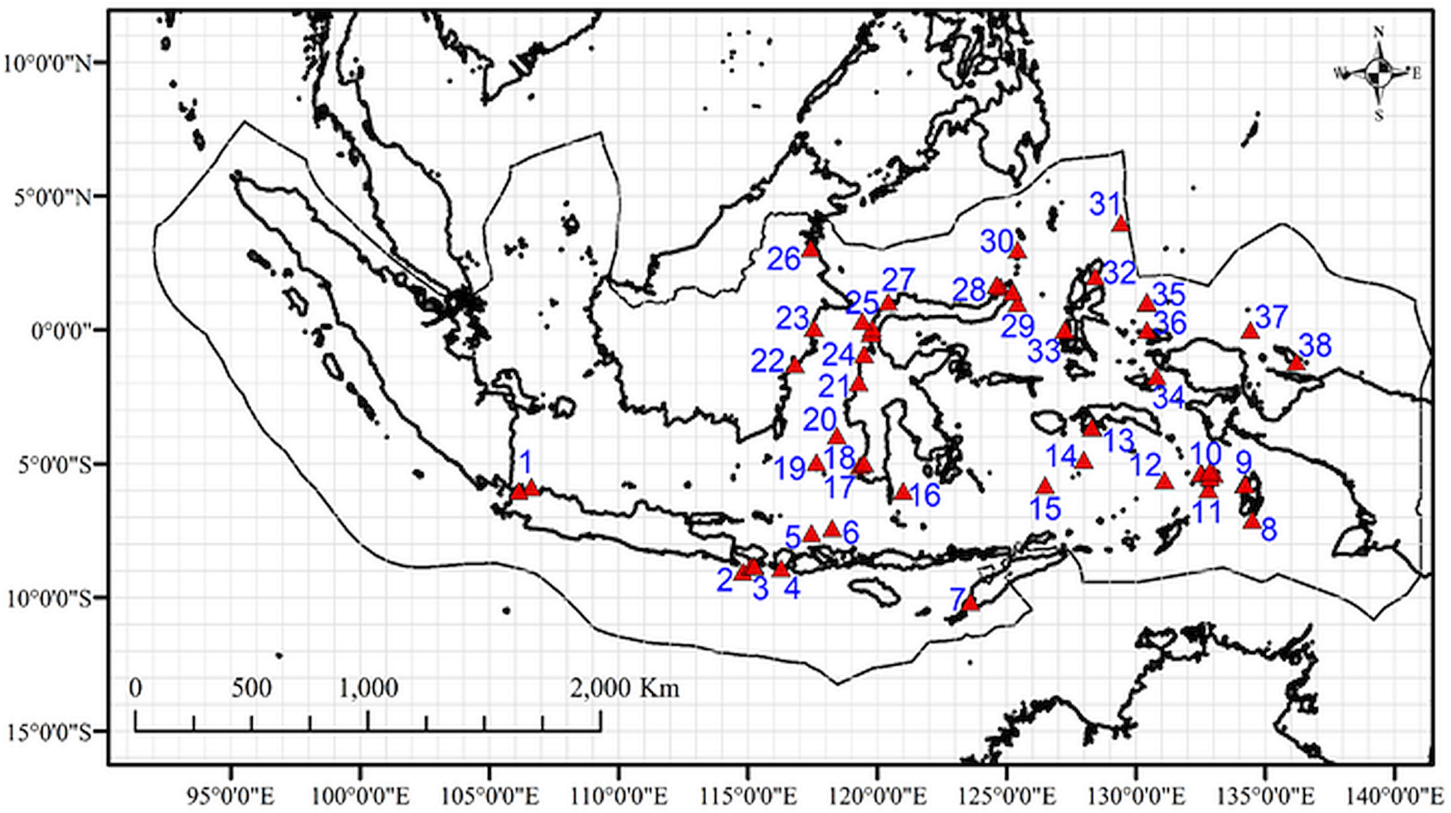
Map of the sites with data on marine amphipod crustaceans (from OBIS, GBIF, published literatures, and field collection) in the Indonesian archipelago overlaid in 1°cells. Numbers represent study sites labels.

### Additional data

Additional data on the geographic distribution of amphipod species were obtained from the Global Biodiversity Information Facility (GBIF Occurrence , 2019), the Ocean Biodiversity Information System (Ocean Biogeographic Information System , 2019), and published literature (Table S2). Data preparation followed the procedure in Arfianti & Costello (2020b). Amphipod occurrences that were mapped outside of the territorial waters of Indonesia were removed using the clip feature in ArcGIS 10.3. We used a global map of amphipod species distributions from Arfianti & Costello (2020b) to classify the occurrence of each benthic species in major oceans (the Arctic, Atlantic, Indian, Pacific, and Antarctic Oceans). Thirty benthic species in Indonesian waters occurred in at least three major oceans (the Pacific, Indian, and Atlantic Oceans) and were therefore classified as “widely distributed” species (Table S3). All pelagic and widely distributed benthic amphipods were excluded because it was already known that they span major biogeographic boundaries (Arfianti & Costello, 2020b) and thus may influence the result if only reported in one part of the study area and not another. After this process, we had a dataset of 116 non-widely distributed benthic species which were mapped and overlaid on 1° × 1° cell grid in ArcGIS 10.3 (Fig. 3). Species occurrences within the same 1° × 1° cell were considered to be the same study site. Therefore, we had 32 sites from additional data (Table S2), and they overlapped with two field sampled sites. Thus, in total, we had a dataset of 147 non-widely distributed benthic species in 38 sites (Fig. 3).

### Data analysis

The presence of 147 non-widespread benthic amphipod crustaceans at 38 sites was used to calculate the similarity of the species composition between all sites using the Jaccard similarity index in PRIMER v7 (Clarke & Gorley, 2015). Cluster analysis using the Jaccard similarity index and group average linkage are commonly used in biogeography and macroecology studies (Costello et al., 2017; Kreft & Jetz, 2010). Importantly, the index is not biased by species absences (Costello et al., 2017). The significance of differences between clusters was determined using the SIMPROF test at *P* < 0.05 (Clarke, Somerfield & Gorley, 2008; Clarke & Gorley, 2015). The similarity percent contribution (SIMPER) of each species within and among groups was also determined. SIMPER identifies species that are most responsible for the observed patterns by disaggregating the Euclidean similarities between samples. We used neural network analysis to ensure the findings were robust to alternative methods of statistical analysis. For this, the dataset was mapped to ‘Infomap Bioregions’ (Edler et al., 2017) and used a minimum latitude-longitude cell size of 1° and a minimum of three species per cell.

Species composition at a site may vary not only due to geographic location but also the sampling methods and habitat sampled. To indicate these factors, the sampling sites were assigned labels based on their data sources (hand collection, light traps, and publications), depth zone, and substrata sampled (Table S2). The nature of cluster analysis means the clusters are the same if we leave out any samples. Thus, omitting samples did not affect the clustering of other samples. While the same two sampling methods (hand collection and light-traps) were used in our field sampling, other sources may have used different field sampling methods and/or sieve sizes. The depth zones were photic (zero-200 m), mesophotic (200–1,000 m), and aphotic deep-sea (more than 1,000 m) following Costello et al. (2018). The seabed substrata were subtidal sediment (literature only), algae and coral rubble (field sampling), and coral reef (light traps only). Very widespread species (including all pelagic amphipods) were omitted from the biogeographic analysis so as to focus on more endemic species (Table S3).

Additional lists of the marine amphipod crustaceans from various regions (tropical and non-tropical) were compiled. These data, latitude midpoint, and seabed area (Exclusive Economic Zones (EEZ) and non-EEZ) from Costello, Cheung & De Hauwere (2010) (Table 3) were plotted to present an objective comparison between the Indonesian amphipod fauna and other regions. The EEZ is a zone where a sovereign state has the right to explore and use marine resources (United Nations, 1982).

**Table 1:** Field study sites location, sampling date, methods, moon phase, and number of taxa sampled. Algae and coral rubble were not sampled at Biak.

**Study site**	**Latitude**	**Longitude**	**Date of sampling**	**Sampling method**	**Moon phase**	**Number of species**
**Ambon**						
	−3.633321	128.325446	21/11/2017	Hand	1st	1
	−3.621758	128.292036	20/11/2017	Light trap	1st	4
**Bali**						
	−8.808450	115.107447	24/01/2018	Hand	1st	6
	−8.792168	115.123170	23/01/2018	Light trap	1st	1
**Balikpapan**						
	−1.281839	116.810687	28/01/2018	Hand	1st	4
	−1.281839	116.810687	27/01/2018	Light trap	1st	2
**Barrang Lompo**						
	−5.051629	119.329406	18/12/2017	Hand	New	3
	−5.050192	119.330313	19/12/2017	Light trap	New	5
**Biak**						
	−1.176280	136.203610	17/11/2017	Light trap	New	4
**Bitung**						
	1.459647	125.233111	14/12/2017	Hand	3rd	7
	1.443100	125.230900	15/12/2017	Light trap	3rd	2
**Dobo**						
	−5.757663	134.223757	22/11/2017	Hand	1st	9
	−5.757663	134.223757	23/11/2017	Light trap	1st	9
**Kupang**						
	−10.149160	123.601550	20/01/2018	Hand	1st	5
	−10.149160	123.601550	20/01/2018	Light trap	1st	7

**Table 2:** Genera and species collected by each sampling method in the field.

**Sampling method and species composition**		
**Hand Collection**	**Light Traps**	**Light Traps and Hand Collection**
*Caprella* Lamarck, 1801	*Ampelisca* Krøyer, 1842 sp. 1	*Ampelisca* Krøyer, 1842 sp. 3
*Cerapus* Say, 1817	*Ampelisca* Krøyer, 1842 sp. 2	*Ampithoe* Leach, 1814
*Gibberosus* cf*. devaneyi* Thomas & Barnard, 1986	*Birubius* Barnard & Drummond, 1976	*Pleusymtes* J.L. Barnard, 1969
*Grandidierella* Coutière, 1904	*Cyproidea liodactyla* Hirayama, 1978	
*Grandifoxus* J.L. Barnard, 1979	*Hyperia macrocephala* (Dana, 1853)	
*Hyale* Rathke, 1836	*Hyperia spinigera* Bovallius, 1889	
*Leucothoe* Leach, 1814	*Hyperia* Latreille, 1823	
*Mallacoota* Barnard, 1972	*Kamaka* Derzhavin, 1923	
*Melita* Leach, 1814	*Lestrigonus* H. Milne Edwards, 1830	
*Parelasmopus dancaui* Ortiz & Lalana, 1997	*Nototropis minikoi* (A.O. Walker, 1905)	
*Pereionotus yongensis* Coleman & Lowry, 2012	*Nototropis* Costa, 1853	
*Photis* Krøyer, 1842	*Orchestia* Leach, 1814	
*Phtisica marina* Slabber, 1769	*Perioculodes* G.O. Sars, 1892	
*Podocerus* Leach, 1814	*Platyischnopus* cf*. mirabilis* Stebbing, 1888	
**	*Rhepoxynius* J.L. Barnard, 1979	
	*Telsosynopia trifidilla* Hughes & Lowry, 2006	
	*Tittakunara* cf. *katoa* Barnard & Drummond, 1979	
	*Urothoe* Dana, 1852	
	*Wandelia orghidani* Ortiz & Lalana, 1997	

## Results

Thirty-six amphipod species from 26 families and 31 genera were recorded from 8 sites during field collection, including the first records of seven families for Indonesia (Table S2). Of these 36 species, four were from the family *Hyperiidae* and one from the family *Lestrigonidae* which are pelagic species and were excluded from the biogeographic analysis (Table S3).

Twenty-two species from 17 genera and 16 families were captured using the light traps. Seventeen species of 17 genera and 15 families were collected by hand from algae and coral rubble. Only three species, each from different genera and families, were recorded from both light traps and hand collection. Thus, the light traps and hand collecting largely collected different species (Table 2).

Light traps in five of the eight sites we sampled (Ambon, Bali, Balikpapan, Biak, and Bitung) collected less than five species (Table 1). The reason for this was unclear and could be due to water movement or low diversity at the sites. The Celebes Sea around Bunaken (site 28) had most species with 45 species, of which 20 species were endemic to Indonesia. Bali, Bontang, and Dobo also had a high number of species with 31, 28, and 17 species, respectively (Table 1 & S2).

**Figure 4:**
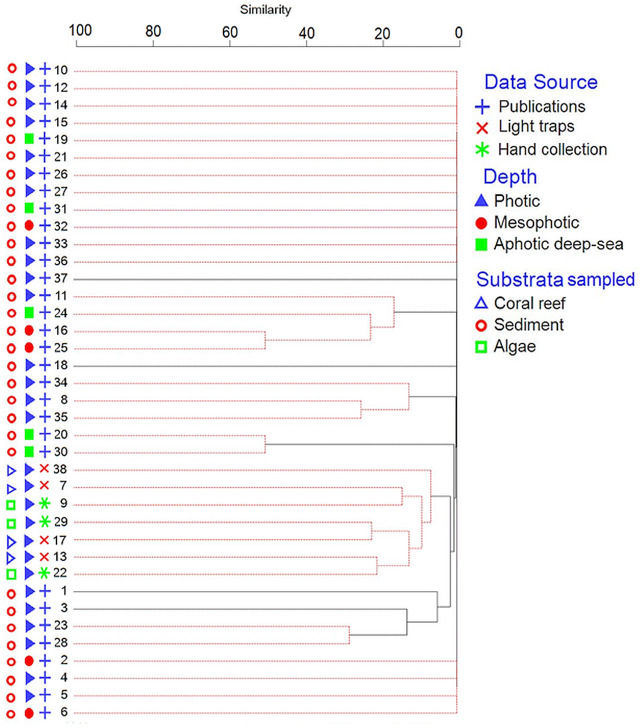
Dendrogram of the similarity of sites using cluster analysis. Red lines represent sites which were not significantly different in species composition by the SIMPROF test (*p* < 0.05). Sampling sites are numbered as in Fig. 3.

The Infomap Bioregions network analysis generated one region for the benthic amphipods (Fig. S1). The hierarchical cluster analysis with the SIMPROF test found five significantly different groups at *P* < 0.05. The main Groups were I (site 11, 16, 24, and 25), II (site 8, 34 and 35), III (site 20 and 30), IV (site 7, 9, 13, 17, 22, 29, and 38), and V (site 1, 3, 23, and 28) (Fig. 4). The 25 species contributed most to the five groups, with *Paradexamine mozambica* (Ledoyer, 1979) and *Onesimoides mindoro* (Lowry & Stoddart, 1993) contributed most to the differences between Group I, Group II, Group III, Group IV, and Group V (Table S4). The groups indicated the sites species composition differed due to sampling method, depth, and substrata. Eighteen sites did not group with any other sites because they had less than five species that were unique to their site (Fig. 4; Table S2). All sites in Groups I, II, III and V were from previously published data (OBIS and literature) and sampled sediment, whereas Group IV sites were from the new field samples (Fig. 4). Group I was from mesophotic depths (except sites 11 and 24), and Group III sites were from aphotic deep-sea. Sites in Groups II and V were from photic depths.

**Figure 5:**
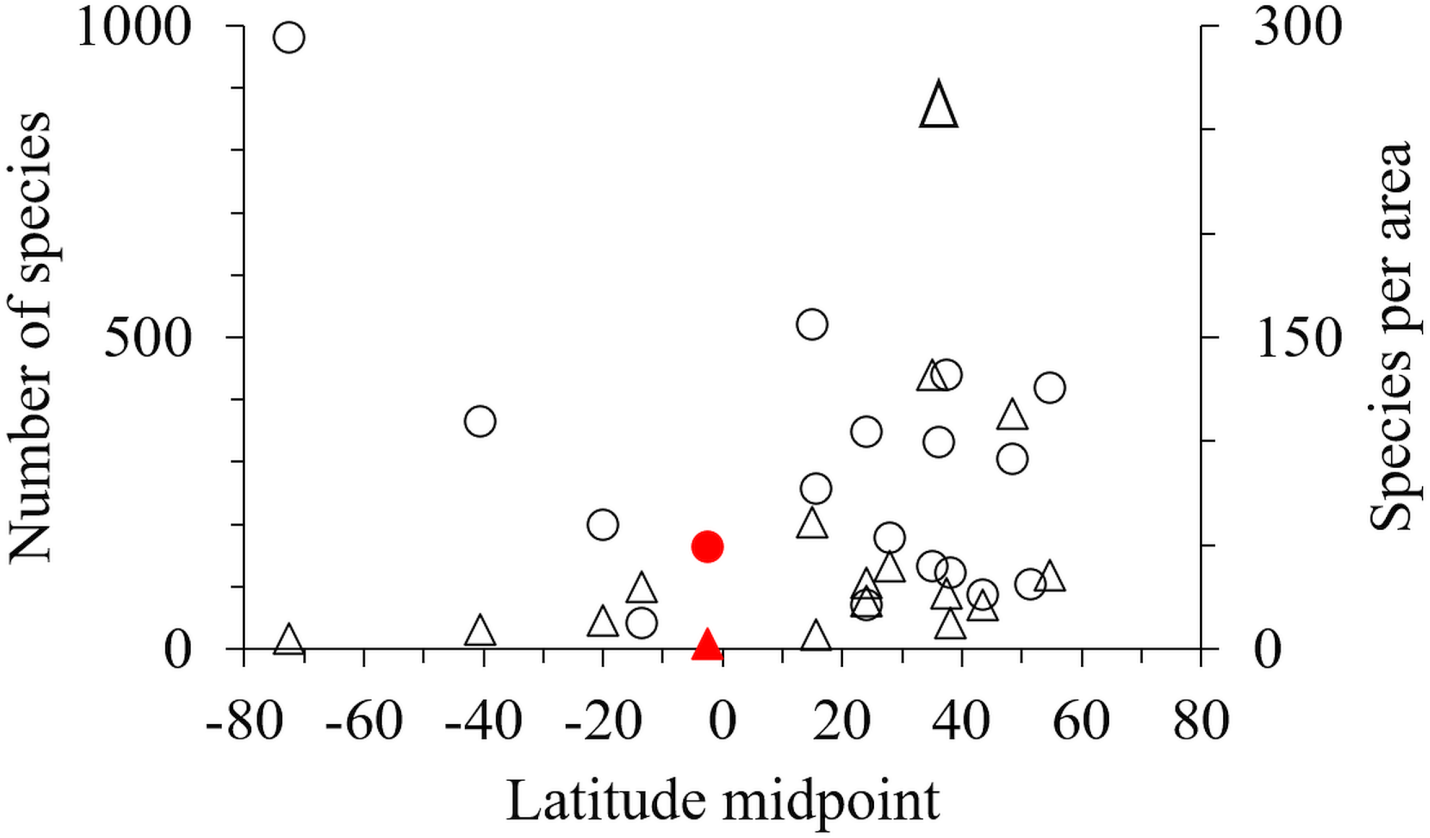
The latitude midpoint of areas with amphipod checklists (Table 3) plotted against species per area (triangle) and number of species (circle). Indonesia has red symbols.

One hundred and seventy-one marine amphipod species have been recorded from Indonesia (Table S2 & Table S3). However, by comparison with 18 other geographic areas Indonesia had fewer species, and fewer species per unit area than most sub-tropical and high latitude areas (Table 3, Fig. 5).

**Table 3:** List of tropical and non-tropical regions with the number of known marine amphipods, latitudinal range, and seabed area. Numbers in bold are the countries’ Exclusive Economic Zones (EEZ) seabed area.

**Region**	**Latitude**	**Area (km^2^)**	**Number of species**	Species* 100,000/ area (km ^**2**^ **)**	**References**
**Tropical**					
Indonesia	6°N–11°S	**5,936,952**	171	3	Present study
The Great Barrier Reef	9°N–22°N	4,036,241	256	6	Lowry & Myers (2009)
New Caledonia	14°S–26°S	**1,423,961**	199	14	Payri & De Forges (2007)
Gulf of Mexico	17°N–31°N	1,541,002	348	23	LeCroy et al. (2009)
Upolu Island, Western Samoa	13°S–14°S	132,181	40	30	Myers (1997)
Pakistan	23°- 25°N	**220,891**	70	32	Bano & Kazmi (2012)
China Seas	3°S–33°N	**855,402**	521	61	Liu (2008)
**Non-Tropical**					
The Southern Ocean	60°S–85°S	20,297,161	(564 Antarctic, 417 sub-Antarctic)	5	De Broyer & Jazdzewska (2014)
New Zealand	28°S–53°S	**4,071,855**	365	9	Webber et al. (2010)
Azores	36°N–40°N	**957,394**	122	13	Bellan-Santini, Marques & Lopes (1993b)
The Black Sea	40°N–47°N	418,412	88	21	Sezgin & Katagan (2007)
Mediterranean Sea	30°N–45°N	1,638,182	441	27	Bellan-Santini et al. (1982) Bellan-Santini et al. (1989) Bellan-Santini et al. (1993a)
Britain and Ireland	49°N–60°N	**1,167,867**	417	36	Costello, Emblow & Picton (1996)
Canary Islands	27°N–29°N	447,890	179	40	Moro et al. (2003)
Gulf of Saint Lawrence	45°N–52°N	268,570	305	114	Brunel, Bossé & Lamarche (1998)
Tunisian coast	33°N–37°N	100,552	133	132	Zakhama-Sraieb, Sghaier & Charfi-Cheikhrouha (2009)
The Algerian Coast	35°N–37°N	127,681	332	260	Bakalem, Dauvin & Grimes (2014)
Lough Hyne	51.5°N	1.2	104	86	Costello & Myers (1991)

## Discussion

For the benthic amphipods analysed, we found one biogeographic region for Indonesia that matched with the global amphipod biogeographic regions proposed by Arfianti & Costello (2020b), studies on reef fish (Mora et al., 2003), corals (Veron et al., 2009), proposed marine ecoregions (Spalding et al., 2007), and previously proposed biogeographic realms based on 65,000 species across all marine taxa (Costello et al., 2017). Previous studies also found no evidence of marine biogeographic boundaries within the region, such as a marine Wallace’s Line (Table S1). Therefore, amphipods do not appear to have a more complex global biogeography than other marine taxa due to their lack of planktonic life stage. This similar global biogeography might be explained by the fact that although amphipods have no planktonic life stage, our and other light-trap samples show many benthic species emerge into the water at night. This nocturnal emergence combined with water currents would aid dispersal of these benthic amphipods. In addition, amphipods may disperse for long-distances as part of fouling communities on floating materials. Globally, at least 108 amphipod species have been reported to passively disperse by such rafting for up to 10,000 km (Thiel & Gutow, 2005; Thiel & Haye, 2006).

The hierarchical cluster analysis showed one group (IV) consisted of all species from our field collection. Other groups consisted of species sampled from sediments using trawls (Pirlot, 1933; Pirlot, 1934; Pirlot, 1936; Pirlot, 1938) and dredges (Gutu, 1997; Ortiz & Lalana, 1997; Ortiz & Lalana, 1999; Ortiz & Lalana, 2003) from the Siboga and Grigore Antipa Expeditions respectively. Thus, previous studies on amphipods in Indonesia mostly sampled sediment using dredges or trawls in contrast to our sampling methods. We found that the species composition of sampling sites was also influenced by the depths sampled (Fig. 4).

The number of species, genera, and families collected from the light traps and hand collections were comparable (Table 2). However, the composition of species and genera was very different, with only 8% of species and 10% of genera in common. Considering the specificity of amphipod species to inhabit particular sediments and hard substrata, including some specialised to live on epifauna and flora, it is to be expected that different sampling method and habitats will find different species (*e.g.*, Burridge et al., 2017; Carvalho et al., 2012; Costello et al., 2016; Costello & Myers, 1987; Costello & Myers, 1991; Crewe, Hamilton & Diamond, 2001; McLeod & Costello, 2017; Michel et al., 2010; Stubbington, Hogan & Wood, 2017; Van Hoey, Degraer & Vincx, 2004; Vázquez-Luis, Sanchez-Jerez & Bayle-Sempere, 2008).

The difference between the light traps and hand collection was the former sampling method collected amphipods which may be challenging to collect by hand due to their mobility or cryptic nature such as *Telsosynopia trifidilla* Hughes & Lowry, 2006, *Hyperia* spp., and *Lestrigonus* spp. which are pelagic amphipods. *Telsosynopia* sp. and other synopiids were previously collected in high abundance (about 100 individuals) using the light traps on the Great Barrier Reef (Hughes, 2009). *Hyperia* and *Lestrigonus* were also collected in light traps in Wellington Harbour, New Zealand (Fincham, 1974) and in zooplankton nets in eastern Australia (Zeidler, 1992). However, the majority of species in our light trap samples were benthic species which emerge into the water at night. They included benthic infaunal burrowing species with fossorial (burrowing) adaptations: *Perioculodes* sp. (Oedicerotidae), *Birubius* sp., *Rhepoxynius* sp. (Phoxocephalidae), *Platyischnopus* sp., *Tittakunara* sp. (Platyischnopidae), and *Urothoe* sp. (Urothoidae) (Table 2). Studies in the Great Barrier Reef and Korean waters using light traps also collected phoxocephalids (*e.g.*, *Birubius kingae* Taylor, 2009, *Birubius oti* Taylor, 2009, *Birubius parvus* Taylor, 2009 and the oedicerotids *Orthomanus koreanus* Kim, Hendrycks & Lee, 2012 and *Imbachoculodes namhaensis* Kim, Hendrycks & Lee, 2012 (Kim, Hendrycks & Lee, 2012; Taylor, 2009). Compared to hand collection, light traps have minimal impact on the environment (McLeod & Costello, 2017), and have proven valuable for collecting a wide diversity of rare and interesting species for museum collections (*e.g.*, Holmes, 1985; Holmes & O’Connor, 1988).

In contrast to the light trap method, hand collection mostly collected epifaunal genera that nestle amongst epibiota (*e.g.*, *Hyale*, *Caprella*, *Leucothoe*, *Mallacoota*, *Melita*, *Parelasmopus*, *Podocerus*) and/or build tubes on hard surfaces (*e.g.*, *Grandidierella*, *Cerapus*, *Photis*) (Table 2). Previous studies also found that *Hyale*, *Mallacoota*, *Melita*, and *Leucothoe* were common among algae, coral rubble, and other epifauna (Barnard, 1965; Kilgallen & Ahyong, 2011; Serejo, 1998). Indeed, *Leucothoe* live inside tunicates and sponges and are best discovered by sampling their hosts (*e.g.*, Costello et al., 1990; Costello & Myers, 1987; Myers & Costello, 1985). On site collection of coral rubble and algae with elutriation by Thomas and co-workers significantly increased the knowledge on leucothoids (*e.g.*, Thomas, 1997; Thomas & Klebba, 2006; Thomas & Klebba, 2007; Thomas & Krapp-Schickel, 2011). In Indonesian waters, *Leucothoe eltoni* Thomas, 2015 was discovered inside tunicates from coral reefs in Raja Ampat (Thomas, 2015).

Our field collection covered only eight sites in the Indonesian archipelago, sampling a limited range of shallow habitats with limited sampling tools. While the inclusion of additional published data expanded these data, particularly into greater depths, we still only reported 171 species in total for the region (Table 3). Although this is an underestimate, Indonesia and other tropical regions with coral reefs seem remarkably low in the number of species per km^2^ compared to non-tropical regions (Table 3, Fig. 5). Considering tropical regions’ status (*e.g.*, Indonesia, The Great Barrier Reef, New Caledonia) as rich-spots of marine biodiversity for corals and fish (Asaad et al., 2018; Jefferson & Costello, 2019), the low number of amphipod species in these regions should be verified by further sampling across a wide range of habitats and depths as possible. However, we hypothesise that low amphipod richness may be due to intense fish predation in the tropics limiting amphipod abundance and diversification. Supporting this hypothesis, fish predation on amphipods is higher in lower latitudes (Reynolds et al., 2018; Whalen et al., 2020) and Edgar et al. (2017) found that while fish dominated shallow coral and rocky reefs in the tropics and sub-tropics, mega-faunal invertebrates increased in diversity in colder high latitudes. Furthermore, a recent study on the richness of habitat forming benthos into which amphipods nestle, including 70,000 species of bivalves, sponges, anthozoans, macroalgae, bryozoans, tube building worms, ascidians, appears depressed in the tropics (M. Pagès-Escolà et al., 2019, unpublished data).

## Conclusions

Our analysis found that amphipod occurrence reflected sampling method, depth, and substrata. Despite Indonesia’s status as one of the biodiversity hotspots in this world, the number of amphipod species reported for Indonesia is notably less than expected. Further sampling should test our hypothesis that amphipod species richness may be depressed by fish predation in the region. Future sampling should consider using various methods including light traps, the wide range of depth, and microhabitats sampled.

## Supplemental Information

10.7717/peerj.12054/supp-1 Supplemental Information 1 Results of the Infomap Bioregions analysis showing that all sites with more than two species were mapped to a single biogeographic region

10.7717/peerj.12054/supp-2 Supplemental Information 2 The studies on marine species related to Wallace’s line. Yes/no means that the study did or did not support Wallace’s Line as a biogeographic boundary

10.7717/peerj.12054/supp-3 Supplemental Information 3 The 147 non-widely distributed benthic amphipod species used in the analysis * = first record of the family for Indonesia. 1 = Type locality distributions from Horton et al. (2019), 2 = Ortiz & Lalana (1999), 3 = (Ortiz & Lalana, 1997), 4 = This study, 5 = (Pirlot, 1936), 6 = Galathea II, Danish Deep-Sea Expedition 1950-52, 7 = Richer, DeForges & Bouchet (1998) Benthic species from the tropical Pacific, 8 = (Pirlot, 1934), 9 = (Arfianti & Wongkamhaeng, 2017), 10 = (Pirlot, 1933), 11 = (Lowry & Stoddart, 1993), 12 = (Pirlot, 1938), 13 = Australian Museum Marine Invertebrate Collection, 14 = Museum and Art Gallery of the Northern Territory, 15 = (Ortiz & Lalana, 2003), 16 = (Krapp-Schickel & Myers, 2006), 17 = (Ledoyer, 1979). ‘Sediment’ includes benthic dredge samples with associated epifauna.

10.7717/peerj.12054/supp-4 Supplemental Information 4 The 30 widely distributed benthic and 15 pelagic species that were not included in the biogeographic analysis 2 = Ortiz & Lalana (1999), 3 = (Ortiz & Lalana, 1997), 4 = This study, 5 = (Pirlot, 1936), 7 = Richer, De Forges & Bouchet (1998) Benthic species from the tropical Pacific, 8 = (Pirlot, 1934), 10 = (Pirlot, 1933), 12 = (Pirlot, 1938), 13 = Australian Museum Marine Invertebrate Collection, 14 = Museum and Art Gallery of the Northern Territory, 16 = (Krapp-Schickel & Myers, 2006).

10.7717/peerj.12054/supp-5 Supplemental Information 5 The 25 species contributing most to the differences between the five groups based on the results of the SIMPER (Similarity Percentage) test Mean Group I is the proportion of sites in which a species is present in Group I. Thus, *Onesimoides mindoro* (Lowry & Stoddart, 1993) was only present in Group I. A value of 0 means the species was not present in any of the sites in a group and 1 means species recorded in all sites in a group. The ‘Contribution %’ is the contribution of a species to the differences between groups.

## Additional Information and Declarations

### Data Availability

The following information was supplied regarding data availability: The data is available at figshare: Arfianti, Tri; Costello, Mark (2021): The distribution of benthic amphipod crustaceans. The University of Auckland. Dataset. https://doi.org/10.17608/k6.auckland.14778162.v1
