# Supplementary figures and images for: The distribution of benthic amphipod crustaceans in Indonesian seas
Source: PeerJ. 2021 Aug 30;9:e12054. doi: 10.7717/peerj.12054 (PMC8411938; doi:10.7717/peerj.12054)

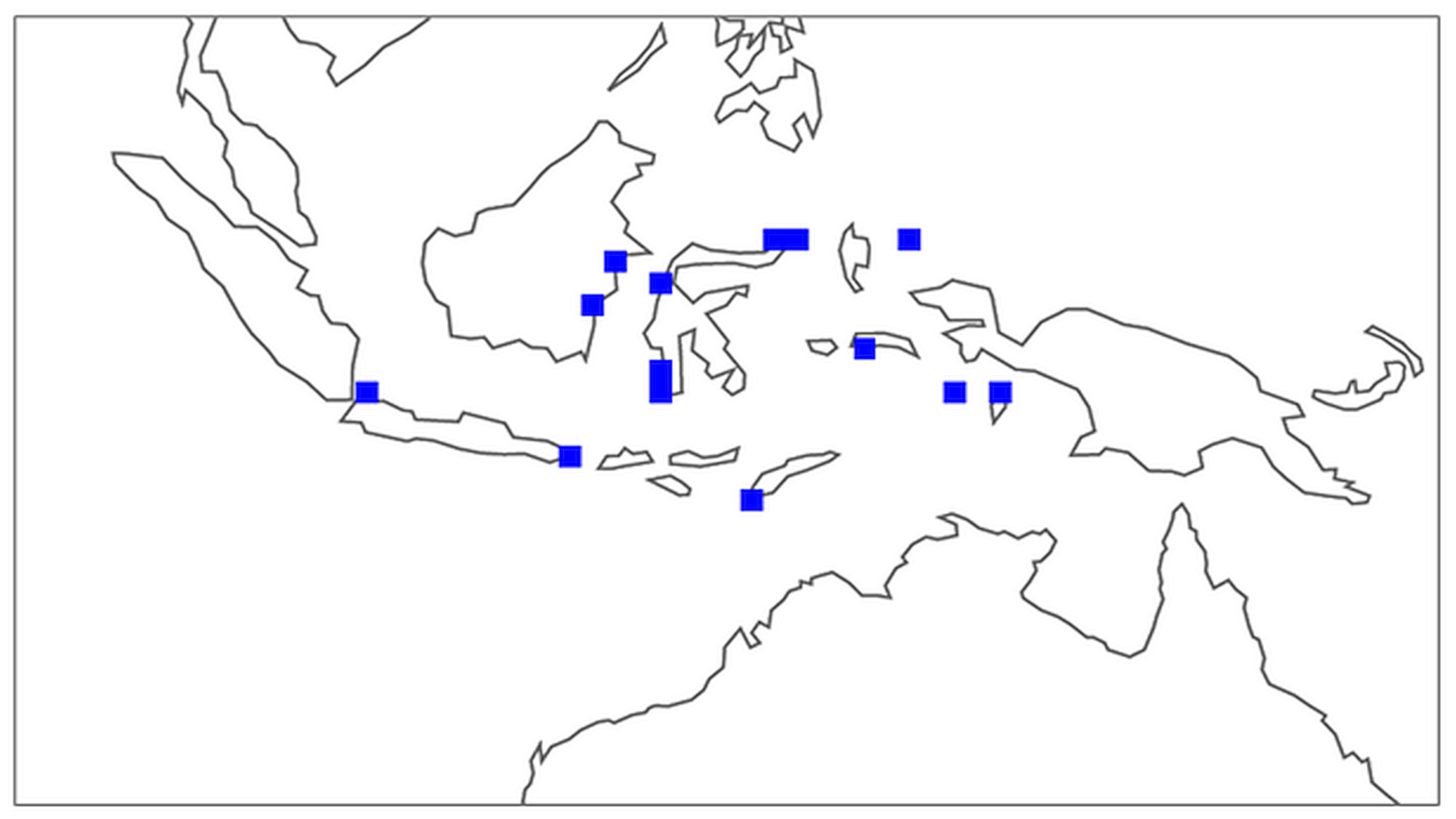

Supplement: Supplemental Information 1 [file peerj-09-12054-s001.png]
