# Supplementary material for: The distribution of benthic amphipod crustaceans in Indonesian seas
Source: PeerJ. 2021 Aug 30;9:e12054. doi: 10.7717/peerj.12054 (PMC8411938; doi:10.7717/peerj.12054)
Supplement: Supplemental Information 2 [file peerj-09-12054-s002.docx]

Table S1. The studies on marine species related to Wallace’s line. Yes/no means that the study did or did not support Wallace’s Line as a biogeographic boundary.

| **Focal study** | **Synthesis** | **Our interpretation** | | **References** |
| --- | --- | --- | --- | --- |
| **Marine Species** |  |  | |  |
| The species distributions of Spanish mackerels (*Scomberomorus*) | The distribution ranges of three species, *S. guttatus*, *S. koreanus* and *S. lineolatu*s stop at the continental margin, at what is known as Wallace's Line | Yes | | Collette & Russo (1984) |
| The species distributions of Staghorn corals (*Acropora*) in Indonesia | The species distribution of Indian and Pacific Ocean *Acropora* overlap within the region | No | | Wallace (2001) |
| The species distributions of 605 zooxanthellate coral | More than 80% of 605 zooxanthellate coral species in the Coral Triangle (CT) region occured in at least 12 of the 16 CT ecoregions | No | | Veron et al., (2009) |
| Distribution patterns of zooxanthellate scleractinia in 4,941 sites and 85 ecoregions worldwide | Global affinities of ecoregions map showed that islands of Wallacea and islands in Sunda and Sahul region are within one province | No | | Veron et al., (2015) |
| Reef fish species dispersal in the Indian and Pacific Ocean | About 86% of the species comprising reef fish assemblages in the Indian and Pacific Oceans were species present in the Indonesian Philippine Region (IPR) | No | | Mora et al., (2003) |
| Populations of *Uroptychus naso* complex (Crustacea: Decapoda: Chirostylidae) | Populations of *U. naso* occured in the Indo-Pacific Ocean | No | | Poore & Andreakis (2011) |
| **Terrestrial Species** |  |  | |  |
| Tetrapods vertebrate zoogeographical regions and subregions | There are three different regions (Indo-Malaysian Region, Wallacean Region, New Guinean Region) for tetrapods vertebrate across Indonesia | Yes | | Procheş & Ramdhani (2012) |
| Biogeography of the 69 species *Hylarana* frog (Anura: Ranidae) across tropical Australasia, Southeast Asia, and Africa | There are three biogeographic regions for *Hylarana* frog across Indonesia that Sulawesi and Southeast Asia (Myanmar, Thailand, Cambodia, Vietnam, Laos, China, Malaysia, Java, Sumatra) is a distinct region | Yes | Oliver et al., (2015) | |
| Distribution data of 1,863 terrestrial snail species from 28 islands and Peninsular Malaysia | Wallacea is not a distinct biogeographic region | No | | Hausdorf (2019) |
| 7,340 species of 896 genera and 165 families of indigenous angiosperms and orchid | Cluster analysis and Principal Component Analysis (PCA) showed that floral composition of indigenous angiosperms and orchid in Java (Sunda Shelf) is similar to Wallacea region | No | | Van Welzen et al., (2011) |
| Geographical distribution of the ant genus *Crematogaster* Lund subgenus *Orthocrema* Santschi in Asia | *C. frtizi* occurred in Peninsular Malaysia, Sumatra, Borneo, and Sulawesi | No | | Hosoishi & Ogata (2016) |
| The biogeographical analysis of *Ficus* subsect. *Urostigma* | Several species of *Ficus* subsect. *Urostigma* crossed Wallace's Line [*F. caulocarpa* (Miq.) Miq., *F. geniculata* Kurz var. *insignis* (Kurz) C.C.Berg, *F. glabella* Blume, *F. prasinicarpa* Elmer ex C.C. Berg, *F. superba* (Miq.) Miq. and *F. virens* Aiton] | No | | Chantarasuwan et al., (2016) |
| Dispersal routes of woody angiosperm genus (*Aglaia*, Meliaceae) across the Indo-Australian Archipelago | *Aglaia* dispersed between the Sunda Shelf and Wallacea and to the Pacific islands, crossing both Wallace's Line and Lydekker's Line. | No | | Grudinski et al., (2014) |
| Data on the distributions and phylogenetic relationships of 21,037 species of amphibians, birds, mammals | Islands in the Wallacea region and islands in Sundaland are in the same Oriental Realm | No | | Holt et al., (2013) |
| 202 Trichoptera species | The well-known Wallace’s line does not act as a faunistic border between Bali and Lombok for Trichoptera | No | | Malicky et al., (2014) |
| Distribution data of 140,425 lichens | Wallace’s line is not a significant dispersal boundary for lichens | No | | Arcadia (2013) |

**References**

**Arcadia LI. 2013.** Lichen biogeography at the largest scales. The Lichenologist 45(4):565–578 DOI 10.1017/S0024282913000170.

**Chantarasuwan B, Rłnsted N, Kjellberg F, Sungkaew S, Van Welzen PC. 2016.** Palaeotropical intercontinental disjunctions revisited using a dated phylogenetic hypothesis with nearly complete species level sampling of Ficus subsect. Urostigma (Moraceae). Journal of Biogeography 43(2):384–397 DOI 10.1111/jbi.12637.

**Collette BB, Russo JL. 1984.** Morphology, systematics, and biology of the Spanish mackerels (Scomberomorus, Scombridae). Fishery Bulletin 82:545–692.

**Grudinski M, Wanntorp L, Pannell CM, Muellner-Riehl AN. 2014.** West to east dispersal in a widespread animal-dispersed woody angiosperm genus (Aglaia, Meliaceae) across the Indo-Australian Archipelago. Journal of Biogeography 41(6):1149–1159 DOI 10.1111/jbi.12280.

**Hausdorf B. 2019.** Beyond Wallace’s line-dispersal of Oriental and Australo-Papuan land-snails across the Indo-Australian Archipelago. Zoological Journal of the Linnean Society 185(1):66–76 DOI 10.1093/zoolinnean/zly031.

**Holt BG, Lessard JP, Borregaard MK, Fritz SA, Araœjo MB, Dimitrov D, Fabre PH, Graham CH, Graves GR. 2013.** An update of Wallace’s zoogeographic regions of the world. Science 339(6115):74–78 DOI 10.1126/science.1228282.

**Hosoishi S, Ogata K. 2016.** Systematics and biogeography of the ant genus Crematogaster Lund subgenus Orthocrema Santschi in Asia (Hymenoptera: Formicidae). Zoological Journal of the Linnean Society 176(3):547–606 DOI 10.1111/zoj.12330.

**Malicky H, Ivanov V, Melnitsky S. 2014.** Caddisflies (Trichoptera) from Lombok. Bali and Java (Indonesia), with a discussion of Wallace’s line. Deutsche Entomologische Zeitschrift 61(1):3–14 DOI 10.3897/dez.61.7046.

**Mora C, Chittaro PM, Sale PF, Kritzer JP, Ludsin SA. 2003.** Patterns and processes in reef fish diversity. Nature 421(6926):933–936 DOI 10.1038/nature01393.

**Oliver LA, Prendini E, Kraus F, Raxworthy CJ. 2015.** Systematics and biogeography of the Hylarana frog (Anura: Ranidae) radiation across tropical Australasia, Southeast Asia, and Africa. Molecular Phylogenetics and Evolution 90:176–192 DOI 10.1016/j.ympev.2015.05.001.

**Poore GC, Andreakis N. 2011.** Morphological, molecular and biogeographic evidence support two new species in the Uroptychus naso complex (Crustacea: Decapoda: Chirostylidae). Molecular Phylogenetics and Evolution 60(1):152–169 DOI 10.1016/j.ympev.2011.03.032.

**Proches Ş, Ramdhani S. 2012.** The world’s zoogeographical regions confirmed by crosstaxon analyses. Bioscience 62(3):260–270 DOI 10.1525/bio.2012.62.3.7.

**Van Welzen PC, Parnell JA, Slik JF. 2011.** Wallace’s Line and plant distributions: two or three phytogeographical areas and where to group Java? Biological Journal of the Linnean Society 103(3):531–545 DOI 10.1111/j.1095-8312.2011.01647.x.

**Veron JE, Devantier LM, Turak E, Green AL, Kininmonth S, Stafford-Smith M, Peterson N. 2009.** Delineating the coral triangle, Galaxea. Journal of Coral Reef Studies 11(2):91–100.

**Veron J, Stafford-Smith M, De Vantier L, Turak E. 2015.** Overview of distribution patterns of zooxanthellate Scleractinia. Frontiers in Marine Science, 1 81:1–19 DOI 10.3389/fmars.2014.00081.

**Wallace CC, Metcalfe I, Smith JMB, Morwood M, Davidson I. 2001.** Wallace’s line and marine organisms: the distribution of staghorn corals (Acropora) in Indonesia. Faunal and Floral Migrations and Evolution in Se Asia Australasia: Rotterdam, Swets and Zeitlinger 171–181.
