# Supplementary material for: The distribution of benthic amphipod crustaceans in Indonesian seas
Source: PeerJ. 2021 Aug 30;9:e12054. doi: 10.7717/peerj.12054 (PMC8411938; doi:10.7717/peerj.12054)
Supplement: Supplemental Information 3 — * = first record of the family for Indonesia. 1 = Type locality distributions from Horton et al. (2019), 2 = Ortiz & Lalana (1999), 3 = (Ortiz & Lalana, 1997), 4 = This study, 5 = (Pirlot, 1936), 6 = Galathea II, Danish Deep-Sea Expedition 1950-52, 7 = Richer, DeForges & Bouchet (1998) Benthic species from the tropical Pacific, 8 = (Pirlot, 1934), 9 = (Arfianti & Wongkamhaeng, 2017), 10 = (Pirlot, 1933), 11 = (Lowry & Stoddart, 1993), 12 = (Pirlot, 1938), 13 = Australian Museum Marine Invertebrate Collection, 14 = Museum and Art Gallery of the Northern Territory, 15 = (Ortiz & Lalana, 2003), 16 = (Krapp-Schickel & Myers, 2006), 17 = (Ledoyer, 1979). ‘Sediment’ includes benthic dredge samples with associated epifauna. [file peerj-09-12054-s003.docx]

Table S2. The 147 non-widely distributed benthic amphipod species used in the analysis. * = first record of the family for Indonesia. 1 = Type locality distributions from Horton et al. (2019), 2 = Ortiz and Lalana (1999), 3 = Ortiz and Lalana, 1997, 4 = This study, 5 = Pirlot, 1936, 6 = Galathea II, Danish Deep-Sea Expedition 1950-52, 7 = Richer De Forges and Bouchet (1998) Benthic species from the tropical Pacific, 8 = Pirlot, 1934, 9 = Arfianti and Wongkamhaeng, 2017, 10 = Pirlot, 1933, 11 = Lowry and Stoddart, 1993, 12 = Pirlot, 1938, 13 = Australian Museum Marine Invertebrate Collection, 14 = Museum and Art Gallery of the Northern Territory, 15 = Ortiz and Lalana, 2003, 16 = Krapp-Schickel and Myers, 2006, 17 = Ledoyer, 1979. ‘Sediment’ includes benthic dredge samples with associated epifauna.

| **Scientific name** | **Site** | **Depth sampled** | **Substrata sampled** | **Sampling method** | **Region** | **Endemic status in Indonesia** | **Data source** |
| --- | --- | --- | --- | --- | --- | --- | --- |
| **Amaryllididae** |  |  |  |  |  |  |  |
| *Bathyamaryllis perezii* Pirlot, 1933 | 11 | Mesophotic | Sediment | Unknown | Wallacea | No | 1 |
| **Ampeliscidae** |  |  |  |  |  |  |  |
| *Ampelisca miops* K.H. Barnard, 1916 | 23 | Photic | Sediment | Dredges | Sunda | No | 2 |
| *Ampelisca pygmaea* Schellenberg, 1938 | 3, 28 | Photic | Sediment | Dredges | Sunda, Wallacea | No | 3 |
| *Ampelisca* sp. 1 | 17 | Photic | Coral reef | Light traps | Wallacea | No | 4 |
| *Ampelisca* sp. 2 | 9 | Photic | Coral reef | Light traps | Sahul | No | 4 |
| *Ampelisca* sp. 3 | 7 | Photic | Coral reef | Light traps | Wallacea | No | 4 |
| *Ampelisca subbrevicornis* Pirlot, 1936 | 28 | Photic | Sediment | Dredges | Wallacea | Yes | 3 |
| *Ampelisca monodi* Ledoyer, 1979 | 13 | Photic | Algae | Unknown | Wallacea | Yes | 17 |
| *Byblis crenulata* Pirlot, 1936 | 35 | Photic | Sediment | Trawl | Sahul | Yes | 5 |
| *Byblis rhinoceros* Pirlot, 1936 | 28 | Mesophotic | Sediment | Dredges | Wallacea | No | 3 |
| *Byblisoides arcillis* (J.L. Barnard, 1961) | 19 | Aphotic deep-sea | Sediment | Unknown | Wallacea | No | 6 |
| **Amphilochidae** |  |  |  |  |  |  |  |
| *Gitanopsis antipai* Ortiz & Lalana, 1997 | 23, 28 | Photic | Sediment | Unknown, Dredges | Sunda, Wallacea | Yes | 1 |
| *Rostrogitanopsis litoralis* Ortiz & Lalana, 1999 | 3 | Photic | Sediment | Dredges | Sunda | Yes | 2 |
| **Ampithoidae** |  |  |  |  |  |  |  |
| *Ampithoe alluaudi* Chevreux, 1901 | 28 | Photic | Sediment | Dredges | Wallacea | Yes | 3 |
| *Ampithoe* sp. | 3, 7, 9, 17, 29 | Photic | Algae, Coral reef | Hand collection, Light traps | Sunda, Wallacea, Sahul | No | 4 |
| *Cymadusa imbroglio* Rabindranath, 1972 | 28 | Photic | Sediment | Dredges | Wallacea | No | 3 |
| *Cymadusa ledoyeri* Peart, 2004 | 23, 28 | Photic | Sediment | Dredges | Sunda, Wallacea | No | 3; 2 |
| *Paragrubia vorax* Chevreux, 1901 | 3 | Photic | Sediment | Dredges | Sunda | No | 2 |
| *Pleonexes kulafi* (J.L. Barnard, 1970) | 3 | Photic | Sediment | Dredges | Sunda | No | 2 |
| **Aoridae** |  |  |  |  |  |  |  |
| *Aoroides columbiae* Walker, 1898 | 28 | Photic | Sediment | Dredges | Wallacea | No | 3 |
| *Autonoe seurati* (Chevreux, 1907) | 28 | Photic | Sediment | Dredges | Wallacea | No | 3 |
| *Bemlos palmatus* (Ledoyer, 1972) | 23 | Photic | Sediment | Dredges | Sunda | Yes | 2 |
| *Bemlos subtriangulum* Ortiz & Lalana, 1997 | 28 | Photic | Sediment | Dredges | Wallacea | Yes | 3 |
| *Bemlos clypeatus* Krapp-Schickell & Myers, 2006 | 3 | Photic | Algae | Unknown | Sunda | No | 16 |
| *Bemlos sicus* Krapp-Schickel & Myers, 2006 | 3 | Photic | Algae | Unknown | Sunda | Yes | 16 |
| *Globosolembos indicus* (Ledoyer, 1967) | 23, 28 | Photic | Sediment | Dredges | Sunda, Wallacea | No | 3 |
| *Globosolembos ruffoi* (Myers, 1975) | 28 | Photic | Sediment | Dredges | Wallacea | No | 3 |
| *Grandidierella bispinosa* Schellenberg, 1938 | 18 | Photic | Sediment | Dredges | Wallacea | No | 2 |
| *Grandidierella gilesi* Chilton, 1921 | 18 | Photic | Sediment | Dredges | Wallacea | No | 2 |
| *Grandidierella longidactylus* Ledoyer, 1982 | 28 | Photic | Sediment | Dredges | Wallacea | No | 3 |
| *Grandidierella* sp. | 9, 17, 22, 29 | Photic | Algae, Coral reef | Light traps, Hand collection | Sahul, Wallacea, Sunda | No | 4 |
| *Lembos podoceroides* Walker, 1904 | 23 | Photic | Sediment | Dredges | Sunda | No | 2 |
| **Atylidae*** |  |  |  |  |  |  |  |
| *Nototropis minikoi* (A.O. Walker, 1905) | 7 | Photic | Coral reef | Light traps | Wallacea | No | 4 |
| *Nototropis* sp. | 7 | Photic | Coral reef | Light traps | Wallacea | No | 4 |
| **Calliopiidae** |  |  |  |  |  |  |  |
| *Oradarea shoemakeri* Pirlot, 1934 | 29 | Photic | Sediment | Trawl | Wallacea | Yes | 8 |
| **Caprellidae** |  |  |  |  |  |  |  |
| *Caprella* sp. | 3 | Photic | Algae | Hand collection | Sunda | No | 4 |
| *Metaprotella sandalensis* Mayer, 1898 | 3, 24, 28 | Photic | Sediment | Dredges, Unknown | Sunda, Wallacea | No | 7 |
| *Paedaridium miserum* Mayer, 1903 | 14 | Photic | Sediment | Unknown | Wallacea | No | 1 |
| *Propodalirius insolitus* Mayer, 1903 | 21 | Photic | Sediment | Unknown | Wallacea | Yes | 7 |
| *Protomima imitatrix* Mayer, 1903 | 23, 28 | Photic | Sediment | Dredges | Sunda, Wallacea | No | 3 |
| **Cheirocratidae** |  |  |  |  |  |  |  |
| *Incratella carpolobata* Ortiz & Lalana, 1999 | 3 | Photic | Sediment | Dredges | Sunda | Yes | 2 |
| **Cheluridae** |  |  |  |  |  |  |  |
| *Tropichelura insulae* (Calman, 1910) | 23, 28 | Photic | Sediment | Dredges | Sunda, Wallacea | No | 3 |
| **Colomastigidae** |  |  |  |  |  |  |  |
| *Colomastix lunalilo* J.L. Barnard, 1970 | 28 | Photic | Sediment | Dredges | Wallacea | No | 3 |
| *Colomastix truncatipes* Ledoyer, 1979 | 1 | Photic | Sediment | Dredges | Sunda | No | 2 |
| **Conicostomatidae** |  |  |  |  |  |  |  |
| *Scolopostoma prionoplax* (Monod, 1937) | 3 | Photic | Sediment | Dredges | Sunda | No | 2 |
| **Corophiidae** |  |  |  |  |  |  |  |
| *Cheiriphotis delloyei* Pirlot, 1934 | 6 | Mesophotic | Sediment | Unknown | Wallacea | Yes | 1 |
| *Cheiriphotis durbanensis* K.H. Barnard, 1916 | 3 | Photic | Sediment | Dredges | Sunda | No | 2 |
| *Cheiriphotis quadrichelatus* Ortiz & Lalana, 1997 | 28 | Photic | Sediment | Unknown | Wallacea | Yes | 1 |
| *Leptocheirus makassarensis* Ortiz & Lalana, 1997 | 23, 28 | Photic | Sediment | Dredges | Sunda, Wallacea | Yes | 3 |
| *Cheiriphotis durbanensis* K.H. Barnard, 1916 | 3 | Photic | Algae | Unknown | Sunda | No | 16 |
| **Cyproideidae** |  |  |  |  |  |  |  |
| *Cyproidea liodactyla* Hirayama, 1978 | 13 | Photic | Coral reef | Light traps | Wallacea | No | 4 |
| *Cyproidea ornata* Haswell, 1879 | 1 | Photic | Sediment | Dredges | Sunda | No | 2 |
| **Dexaminidae** |  |  |  |  |  |  |  |
| *Dexaminoculus lacinimanus* Ortiz & Lalana, 1999 | 23 | Photic | Sediment | Dredges | Sunda | Yes | 2 |
| *Guernea (Guernea) brevispinis* Ledoyer, 1982 | 23 | Photic | Sediment | Dredges | Sunda | No | 2 |
| *Guernea (Guernea) spinicornis* Ledoyer, 1982 | 28 | Photic | Sediment | Dredges | Wallacea | No | 3 |
| *Guernea sulawesiensis* Ortiz & Lalana, 1997 | 23, 28 | Photic | Sediment | Dredges, Unknown | Sunda, Wallacea | Yes | 1 |
| *Paradexamine micronesica* Ledoyer, 1978 | 1, 23, 28 | Photic | Sediment | Dredges | Sunda, Wallacea | No | 3 |
| *Paradexamine mozambica* Ledoyer, 1979 | 1, 3, 23, 28 | Photic | Sediment | Dredges | Sunda, Wallacea | No | 3 |
| *Paradexamine orientalis* (Spandl, 1923) | 18, 28 | Photic | Sediment | Dredges | Wallacea | Yes | 3 |
| *Paradexamine serraticra* (Walker, 1904) | 28 | Photic | Sediment | Dredges | Wallacea | Yes | 3 |
| *Polycheria atolli* Walker, 1905 | 3 | Photic | Sediment | Dredges | Sunda | No | 2 |
| **Eophliantidae** |  |  |  |  |  |  |  |
| *Wandelia orghidani* Ortiz & Lalana, 1997 | 7, 28 | Photic | Coral reef | Light traps, Unknown | Wallacea | Yes | 1 |
| **Eriopisidae** |  |  |  |  |  |  |  |
| *Eriopisella paraupolu* Ortiz & Lalana, 1997 | 23 | Photic | Sediment | Dredges | Sunda | Yes | 3 |
| *Maleriopa dentifera* (Ledoyer, 1978) | 3 | Photic | Sediment | Dredges | Sunda | Yes | 2 |
| *Psammogammarus wallacei* Vonk, Hoeksema & Jaume, 2011 | 33 | Photic | Sediment | Unknown | Wallacea | Yes | 1 |
| *Victoriopisa bantenensis* Arfianti & Wongkamhaeng, 2017 | 1 | Photic | Sediment | Dredges | Sunda | Yes | 9 |
| **Hornelliidae** |  |  |  |  |  |  |  |
| *Hornellia (Hornellia) incerta* Walker, 1904 | 23 | Photic | Sediment | Dredges | Sunda | No | 2 |
| **Hyalidae*** |  |  |  |  |  |  |  |
| *Hyale* sp. | 3, 9, 13, 22, 29 | Photic | Algae | Hand collection | Sunda, Sahul, Wallacea | No | 4 |
| **Ischyroceridae** |  |  |  |  |  |  |  |
| *Cerapus* sp. | 9, 22 | Photic | Algae | Hand collection | Sahul, Sunda | No | 4 |
| *Ericthonius pugnax* (Dana, 1852) | 3, 23, 28 | Photic | Sediment | Dredges | Sunda, Wallacea | No | 3 |
| **Kamakidae** |  |  |  |  |  |  |  |
| *Aorchoides dilatata* Ledoyer, 1972 | 28 | Photic | Sediment | Dredges | Wallacea | Yes | 3 |
| *Kamaka* sp. | 9 | Photic | Coral reef | Light traps | Sahul | No | 4 |
| *Kamaka taditadi* Thomas & Barnard, 1991 | 4 | Photic | Algae | Unknown | Wallacea | No | 16 |
| **Lepechinellidae** |  |  |  |  |  |  |  |
| *Lepechinella curvispinosa* Pirlot, 1933 | 32 | Mesophotic | Sediment | Trawl | Wallacea | Yes | 10 |
| *Paralepechinella longipalpa* Pirlot, 1933 | 25 | Aphotic deep-sea | Sediment | Trawl | Wallacea | Yes | 10 |
| **Leucothoidae** |  |  |  |  |  |  |  |
| *Anamixis stebbingi* Walker, 1904 | 3 | Photic | Sediment | Dredges | Sunda | No | 2 |
| *Leucothoe bannwarthi* (Schellenberg, 1928) | 23 | Photic | Sediment | Dredges | Sunda | No | 3 |
| *Leucothoe crenatipalma* Ledoyer, 1972 | 3 | Photic | Sediment | Dredges | Sunda | No | 2 |
| *Leucothoe dentata* Ledoyer, 1973 | 3, 23, 28 | Photic | Sediment | Dredges | Sunda, Wallacea | No | 3 |
| *Leucothoe furina* (Savigny, 1816) | 15 | Photic | Sediment | Trawl | Wallacea | No | 4 |
| *Leucothoe hyhelia* J.L. Barnard, 1965 | 3, 28 | Photic | Sediment | Dredges | Sunda, Wallacea | No | 3 |
| *Leucothoe madrasana* Sivaprakasam, 1969 | 23, 28 | Photic | Sediment | Dredges | Sunda, Wallacea | No | 3 |
| *Leucothoe micronesiae* J.L. Barnard, 1965 | 28 | Photic | Sediment | Dredges | Wallacea | No | 3 |
| *Leucothoe predenticulata* Ledoyer, 1978 | 3, 23 | Photic | Sediment | Dredges | Sunda | Yes | 2 |
| *Leucothoe* sp. | 9, 29 | Photic | Algae | Hand collection | Sahul, Wallacea | No | 4 |
| *Paranamixis ledoyeri* Ortiz & Lalana, 1997 | 28 | Photic | Sediment | Unknown | Wallacea | Yes | 1 |
| **Lysianassidae** |  |  |  |  |  |  |  |
| *Azotostoma bunakenensis* Ortiz & Lalana, 1997 | 28 | Photic | Sediment | Dredges | Wallacea | Yes | 3 |
| *Charcotia enoei* (Stephensen, 1931) | 8, 35 | Photic | Sediment | Unknown, Trawl | Sahul | No | 1 |
| *Charcotia selayarensis (*Lowry & Kilgallen, 2014) | 37 | Photic | Sediment | Trawl | Sahul | No | 5 |
| *Socarnes tuscarora* Lowry & Stoddart, 1994 | 11 | Photic | Sediment | Unknown | Wallacea | No | 7 |
| **Maeridae** |  |  |  |  |  |  |  |
| *Bathyceradocus stephenseni* Pirlot, 1934 | 29 | Aphotic deep-sea | Sediment | Trawl | Wallacea | No | 8 |
| *Ceradocus (Denticeradocus) serratus* (Spence Bate, 1862) | 23 | Mesophotic | Sediment | Dredges | Sunda | No | 3 |
| *Elasmopus gracilis* Schellenberg, 1938 | 23 | Photic | Sediment | Dredges | Sunda | No | 3 |
| *Elasmopus hooheno* J.L. Barnard, 1970 | 3 | Photic | Sediment | Dredges | Sunda | No | 2 |
| *Mallacoota odontoplax* (Pirlot, 1936) | 5 | Photic | Sediment | Unknown | Wallacea | Yes | 1 |
| *Mallacoota* sp. | 3, 9, 17 | Photic | Algae | Hand collection | Sunda, Sahul, Wallacea | No | 4 |
| *Mallacoota subcarinata* (Haswell, 1879) | 27 | Photic | Sediment | Trawl | Wallacea | No | 5 |
| *Parelasmopus dancaui* Ortiz & Lalana, 1997 | 1, 7, 28 | Photic | Sediment, Algae | Dredges, Hand collection, Unknown | Sunda, Wallacea | No | 1 |
| *Parelasmopus setiger* Chevreux, 1901 | 23, 28 | Photic | Sediment | Dredges | Sunda, Wallacea | No | 3 |
| *Parelasmopus suluensis* (Dana, 1852) | 35 | Photic | Sediment | Trawl | Sahul | No | 5 |
| *Quadrimaera serrata* (Schellenberg, 1938) | 3, 23, 28 | Photic | Sediment | Dredges | Sunda, Wallacea | No | 3 |
| **Megaluropidae*** |  |  |  |  |  |  |  |
| *Gibberosus* cf*. devaneyi* Thomas & Barnard, 1986 | 9 | Photic | Algae | Hand collection | Sahul | No | 4 |
| **Melitidae** |  |  |  |  |  |  |  |
| *Dulichiella fresnelii* (Audouin, 1826) | 26 | Photic | Sediment | Trawl | Sunda | No | 5 |
| *Melita* sp*.* | 7, 17 | Photic | Algae | Hand collection | Wallacea | No | 4 |
| **Oedicerotidae** |  |  |  |  |  |  |  |
| *Perioculodes* sp. | 17, 22 | Photic | Coral reef | Light traps | Sunda, Wallacea | No | 4 |
| **Pakynidae** |  |  |  |  |  |  |  |
| *Figorella corindon* Lowry & Stoddart, 1993 | 24 | Aphotic deep-sea | Sediment | Unknown | Wallacea | Yes | 7 |
| **Paracalliopiidae** |  |  |  |  |  |  |  |
| *Katocalliope gutui* Ortiz & Lalana, 1997 | 28 | Photic | Sediment | Dredges | Wallacea | Yes | 3 |
| *Paracalliope bacescui* Ortiz & Lalana, 1997 | 28 | Photic | Sediment | Dredges | Wallacea | Yes | 3 |
| **Phliantidae*** |  |  |  |  |  |  |  |
| *Pereionotus yongensis* Coleman & Lowry, 2012 | 9 | Photic | Algae | Hand collection | Sahul | No | 4 |
| **Photidae** |  |  |  |  |  |  |  |
| *Ampelisciphotis tridens* Pirlot, 1938 | 3, 28, 34, 35 | Photic | Sediment | Dredges, Unknown, Trawl | Sunda, Wallacea, Sahul | No | 1 |
| *Dodophotis distinguenda* (Ruffo, 1955) | 28 | Photic | Sediment | Dredges | Wallacea | Yes | 3 |
| *Photis cavimana* Ledoyer, 1979 | 3, 28 | Photic | Sediment | Dredges | Sunda, Wallacea | Yes | 3 |
| *Photis* sp*.* | 9, 29 | Photic | Algae | Hand collection | Sahul, Wallacea | No | 4 |
| **Phoxocephalidae** |  |  |  |  |  |  |  |
| *Baliphoxus andresi* Ortiz & Lalana, 1999 | 3 | Photic | Sediment | Dredges | Sunda | Yes | 2 |
| *Birubius bali* Ortiz & Lalana, 1999 | 3 | Photic | Sediment | Dredges | Sunda | Yes | 2 |
| *Birubius murariui* Ortiz & Lalana, 1997 | 23, 28 | Photic | Sediment | Dredges | Sunda, Wallacea | Yes | 3 |
| *Birubius* sp*.* | 7, 9 | Photic | Coral reef | Light traps | Sahul, Wallacea | No | 4 |
| *Grandifoxus* sp*.* | 29 | Photic | Algae | Hand collection | Wallacea | No | 4 |
| *Harpiniopsis spaercki* (Dahl, 1959) | 12 | Photic | Sediment | Unknown | Wallacea | Yes | 6 |
| *Pseudharpinia abyssalis* (Pirlot, 1932) | 2 | Mesophotic | Sediment | Unknown | Sunda | No | 6 |
| *Rhepoxynius* sp*.* | 9 | Photic | Coral reef | Light traps | Sahul | No | 4 |
| **Platyischnopidae*** |  |  |  |  |  |  |  |
| *Platyischnopus* cf*. mirabilis* Stebbing, 1888 | 9 | Photic | Coral reef | Light traps | Sahul | No | 4 |
| *Tittakunara* cf. *katoa* Drummond, 1979 | 9 | Photic | Coral reef | Light traps | Sahul | No | 4 |
| **Pleustidae*** |  |  |  |  |  |  |  |
| *Pleusymtes* sp*.* | 3, 9, 13, 22 | Photic | Coral reef | Light traps | Sunda, Sahul, Wallacea | No | 4 |
| **Podoceridae** |  |  |  |  |  |  |  |
| *Podocerus* sp*.* | 3, 7, 9 | Photic | Algae | Hand collection | Sunda, Sahul, Wallacea | No | 4 |
| *Podocerus walkeri walkeri* Rabindranath, 1972 | 28 | Photic | Sand | Dredges | Wallacea | No | 15 |
| **Pontogeneiidae** |  |  |  |  |  |  |  |
| *Tethygeneia pacifica* (Schellenberg, 1938) | 23 | Photic | Sediment | Dredges | Sunda | No | 2 |
| **Stenothoidae** |  |  |  |  |  |  |  |
| *Metopa abyssi* Pirlot, 1933 | 29 | Aphotic deep-sea | Sediment | Trawl | Wallacea | Yes | 10 |
| **Synopiidae** |  |  |  |  |  |  |  |
| *Pseudotiron longicaudatus* Pirlot, 1934 | 32 | Mesophotic | Sediment | Trawl | Wallacea | Yes | 8 |
| *Telsosynopia trifidilla* Hughes & Lowry, 2006 | 13, 17, 38 | Photic | Coral reef | Light traps | Wallacea, Sahul | No | 4 |
| *Telsosynopia paravariabilis* (Ortiz & Lalana, 1997) | 3, 23, 28 | Photic | Sediment | Dredges | Sunda, Wallacea | Yes | 3 |
| **Talitridae** |  |  |  |  |  |  |  |
| *Orchestia* sp. | 7, 13 | Photic | Coral reef | Light traps | Wallacea | No | 4 |
| *Talorchestia bunaken* Lowry, Springthorpe & Azman, 2017 | 28 | Photic | Sediment | Unknown | Wallacea | Yes | 1 |
| *Talorchestia mindorensis* Oleröd, 1970 | 18 | Photic | Sediment | Dredges | Wallacea | No | 2 |
| *Talorchestia yoyoae* Lowry, Springthorpe & Azman, 2017 | 4 | Photic | Sediment | Unknown | Wallacea | Yes | 1 |
| **Tryphosidae** |  |  |  |  |  |  |  |
| *Hippomedon bandae* Pirlot, 1933 | 31 | Aphotic deep-sea | Sediment | Trawl | Wallacea | Yes | 10 |
| *Onesimoides castellatus* Lowry & Stoddart, 1993 | 11 | Photic | Sediment | Unknown | Wallacea | No | 7 |
| *Onesimoides chelatus* Pirlot, 1933 | 20, 29, 30 | Aphotic deep-sea | Sediment | Unknown, Trawl | Wallacea | No | 6 |
| *Onesimoides mindoro* Lowry & Stoddart, 1993 | 11, 16, 24, 25 | Mesophotic | Sediment | Unknown | Wallacea | No | 7 |
| *Paracentromedon pacificus* Lowry & Stoddart, 1993 | 24 | Aphotic deep-sea | Sediment | Unknown | Wallacea | Yes | 7 |
| *Paronesimoides lignivorus* Pirlot, 1933 | 30 | Aphotic deep-sea | Sediment | Trawl | Wallacea | Yes | 10 |
| *Photosella mucronata* (Pirlot, 1936) | 36 | Photic | Sediment | Trawl | Sahul | No | 5 |
| **Unciolidae** |  |  |  |  |  |  |  |
| *Wombalano basilatissima* (Ortiz & Lalana, 1999) | 3 | Photic | Sediment | Dredges | Sunda | Yes | 2 |
| **Uristidae** |  |  |  |  |  |  |  |
| *Ichnopus annasona* Lowry & Stoddart, 1992 | 10 | Photic | Sediment | Unknown | Wallacea | No | 7 |
| *Ichnopus wardi* Lowry & Stoddart, 1992 | 11 | Photic | Sediment | Unknown | Wallacea | No | 11 |
| **Urothoidae*** |  |  |  |  |  |  |  |
| *Urothoe* sp*.* | 7, 29 | Photic | Coral reef | Light traps | Wallacea | No | 4 |
