# Supplementary material for: The distribution of benthic amphipod crustaceans in Indonesian seas
Source: PeerJ. 2021 Aug 30;9:e12054. doi: 10.7717/peerj.12054 (PMC8411938; doi:10.7717/peerj.12054)
Supplement: Supplemental Information 4 — 2 = Ortiz & Lalana (1999), 3 = (Ortiz & Lalana, 1997), 4 = This study, 5 = (Pirlot, 1936), 7 = Richer, De Forges & Bouchet (1998) Benthic species from the tropical Pacific, 8 = (Pirlot, 1934), 10 = (Pirlot, 1933), 12 = (Pirlot, 1938), 13 = Australian Museum Marine Invertebrate Collection, 14 = Museum and Art Gallery of the Northern Territory, 16 = (Krapp-Schickel & Myers, 2006). [file peerj-09-12054-s004.docx]

Table S3. The 30 widely distributed benthic and 15 pelagic species that were not included in the biogeographic analysis. 2 = Ortiz and Lalana (1999), 3 = Ortiz and Lalana, 1997, 4 = This study, 5 = Pirlot, 1936, 7 = Richer De Forges and Bouchet (1998) Benthic species from the tropical Pacific, 8 = Pirlot, 1934, 10 = Pirlot, 1933, 12 = Pirlot, 1938, 13 = Australian Museum Marine Invertebrate Collection, 14 = Museum and Art Gallery of the Northern Territory, 16 = Krapp-Schickel and Myers, 2006.

| **Scientific name** | **Source** |
| --- | --- |
| **Amaryllididae** |  |
| *Amaryllis macrophthalma* Haswell, 1879 | 10 |
| **Amathillopsidae** |  |
| *Cleonardopsis carinata* K.H. Barnard, 1916 | 8 |
| **Ampeliscidae** |  |
| *Ampelisca tenuicornis* Liljeborg, 1856 | 3; 2 |
| **Amphilochidae** |  |
| *Hourstonius pusilla* (K.H. Barnard, 1916) | 3 |
| **Ampithoidae** |  |
| *Sunamphitoe pelagica* (H. Milne Edwards, 1830) | 3 |
| *Ampithoe ramondi* Audouin, 1826 | 3 |
| *Cymadusa brevidactyla* (Chevreux, 1907) | 3 |
| **Aoridae** |  |
| *Grandidierella bonnieroides* Stephensen, 1947 | 3 |
| *Lembos hypacanthus* K.H. Barnard, 1916 | 16 |
| **Calliopiidae** |  |
| *Harpinioides drepanocheir* Stebbing, 1888 | 8 |
| **Caprellidae** |  |
| *Phtisica marina* Slabber, 1769 | 3 |
| **Cheluridae** |  |
| *Chelura terebrans* Philippi, 1839 | 12 |
| **Corophiidae** |  |
| *Cheiriphotis megacheles* (Giles, 1885) | 12 |
| **Eurytheneidae** |  |
| *Eurythenes gryllus* (Lichtenstein in Mandt, 1822) | 3 |
| *Eurythenes thurstoni* Stoddart & Lowry, 2004 | 7 |
| **Ischyroceridae** |  |
| *Ericthonius brasiliensis* (Dana, 1853) | 3 |
| **Leucothoidae** |  |
| *Leucothoe richiardii* Lessona, 1865 | 3 |
| *Leucothoe spinicarpa* (Abildgaard, 1789) | 3 |
| **Liljeborgiidae** |  |
| *Liljeborgia dubia* (Haswell, 1880) | 5 |
| **Lysianassidae** |  |
| *Lysianassa ceratina* (Walker, 1889) | 2 |
| **Maeridae** |  |
| *Elasmopus pectenicrus* (Spence Bate, 1862) | 13 |
| *Quadrimaera pacifica* (Schellenberg, 1938) | 2 |
| *Ceradocus (Denticeradocus) rubromaculatus* (Stimpson, 1855) | 5 |
| *Dulichiella appendiculata* (Say, 1818) | 3 |
| **Oedicerotidae** |  |
| *Perioculodes aequimanus* (Kossman, 1880) | 3 |
| **Oxycephalidae** |  |
| *Glossocephalus milneedwardsi* Bovallius, 1887 | 12 |
| **Photidae** |  |
| *Latigammaropsis atlantica* (Stebbing, 1888) | 3 |
| *Photis longicaudata* (Spence Bate & Westwood, 1862) | 3 |
| **Pontogeneiidae** |  |
| *Pontogeneia inermis* (Krøyer, 1838) | 14 |
| **Sebidae** |  |
| *Seba typica* (Chilton, 1884) | 3 |
| **Cyphocarididae** |  |
| *Cyphocaris anonyx* Boeck, 1871 | 10 |
| *Cyphocaris challengeri* Stebbing, 1888 | 10 |
| *Cyphocaris faurei* K.H. Barnard, 1916 | 10; 3 |
| **Cystisomatidae** |  |
| *Cystisoma pellucida* (Willemöes-Suhm, 1873) | 12 |
| **Hyperiidae** |  |
| *Hyperia macrocephala* (Dana, 1853) | 4 |
| *Hyperia spinigera* Bovallius, 1889 | 4 |
| *Hyperia* sp. | 4 |
| **Lestrigonidae** |  |
| *Lestrigonus* sp. | 4 |
| **Lycaeidae** |  |
| *Simorhynchotus antennarius* (Claus, 1871) | 12 |
| **Oxycephalidae** |  |
| *Leptocotis tenuirostris* (Claus, 1871) | 12 |
| *Oxycephalus clausi* Bovallius, 1887 | 12 |
| *Oxycephalus latirostris* Claus, 1879 | 12 |
| *Rhabdosoma whitei* Spence Bate, 1862 | 12 |
| *Streetsia challengeri* Stebbing, 1888 | 12 |
| *Calamorhynchus pellucidus* Streets, 1878 | 12 |
