# Supplementary material for: The distribution of benthic amphipod crustaceans in Indonesian seas
Source: PeerJ. 2021 Aug 30;9:e12054. doi: 10.7717/peerj.12054 (PMC8411938; doi:10.7717/peerj.12054)
Supplement: Supplemental Information 5 — Mean Group I is the proportion of sites in which a species is present in Group I. Thus, Onesimoides mindoro (Lowry & Stoddart, 1993) was only present in Group I. A value of 0 means the species was not present in any of the sites in a group and 1 means species recorded in all sites in a group. The ‘Contribution %’ is the contribution of a species to the differences between groups. [file peerj-09-12054-s005.docx]

Table S4. The 25 species contributing most to the differences between the five groups based on the results of the SIMPER (Similarity Percentage) test. Mean Group I is the proportion of sites in which a species is present in Group I. Thus, *Onesimoides mindoro* Lowry & Stoddart, 1993 was only present in Group I. A value of 0 means the species was not present in any of the sites in a group and 1 means species recorded in all sites in a group. The ‘Contribution %’ is the contribution of a species to the differences between groups.

| **Species** | **Contribution %** | **Mean Group I** | **Mean Group II** | **Mean Group III** | **Mean Group IV** | **Mean Group V** |
| --- | --- | --- | --- | --- | --- | --- |
| *Paradexamine mozambica* Ledoyer, 1979 | 2.2 | 0.0 | 0.0 | 0.0 | 0.0 | 1.0 |
| *Onesimoides mindoro* Lowry & Stoddart, 1993 | 2.2 | 1.0 | 0.0 | 0.0 | 0.0 | 0.0 |
| *Hyale* sp. | 2.1 | 0.0 | 0.0 | 0.0 | 0.6 | 0.3 |
| *Ampithoe* sp. | 2.1 | 0.0 | 0.0 | 0.0 | 0.6 | 0.3 |
| *Ampelisciphotis tridens* Pirlot, 1938 | 2.0 | 0.0 | 0.7 | 0.0 | 0.0 | 0.5 |
| *Grandidierella* sp. | 1.8 | 0.0 | 0.0 | 0.0 | 0.6 | 0.0 |
| *Pleusymtes* sp. | 1.7 | 0.0 | 0.0 | 0.0 | 0.4 | 0.3 |
| *Telsosynopia paravariabilis* (Ortiz & Lalana, 1997) | 1.7 | 0.0 | 0.0 | 0.0 | 0.0 | 0.8 |
| *Quadrimaera serrata* (Schellenberg, 1938) | 1.7 | 0.0 | 0.0 | 0.0 | 0.0 | 0.8 |
| *Ericthonius pugnax* (Dana, 1852) | 1.7 | 0.0 | 0.0 | 0.0 | 0.0 | 0.8 |
| *Leucothoe dentata* Ledoyer, 1973 | 1.7 | 0.0 | 0.0 | 0.0 | 0.0 | 0.8 |
| *Paradexamine micronesica* Ledoyer, 1978 | 1.7 | 0.0 | 0.0 | 0.0 | 0.0 | 0.8 |
| *Onesimoides chelatus* Pirlot, 1933 | 1.6 | 0.0 | 0.0 | 1.0 | 0.1 | 0.0 |
| *Metaprotella sandalensis* Mayer, 1898 | 1.5 | 0.3 | 0.0 | 0.0 | 0.0 | 0.5 |
| *Parelasmopus dancaui* Ortiz & Lalana, 1997 | 1.4 | 0.0 | 0.0 | 0.0 | 0.1 | 0.5 |
| *Telsosynopia trifidilla* | 1.4 | 0.0 | 0.0 | 0.0 | 0.4 | 0.0 |
| *Mallacoota* sp. | 1.3 | 0.0 | 0.0 | 0.0 | 0.3 | 0.3 |
| *Podocerus* sp. | 1.3 | 0.0 | 0.0 | 0.0 | 0.3 | 0.3 |
| *Charcotia enoei* (Stephensen, 1931) | 1.2 | 0.0 | 0.7 | 0.0 | 0.0 | 0.0 |
| *Photis cavimana* Ledoyer, 1979 | 1.1 | 0.0 | 0.0 | 0.0 | 0.0 | 0.5 |
| *Protomima imitatrix* Mayer, 1903 | 1.1 | 0.0 | 0.0 | 0.0 | 0.0 | 0.5 |
| *Guernea sulawesiensis* Ortiz & Lalana, 1997 | 1.1 | 0.0 | 0.0 | 0.0 | 0.0 | 0.5 |
| *Globosolembos indicus* (Ledoyer, 1967) | 1.1 | 0.0 | 0.0 | 0.0 | 0.0 | 0.5 |
| *Parelasmopus setiger* Chevreux, 1901 | 1.1 | 0.0 | 0.0 | 0.0 | 0.0 | 0.5 |
| *Birubius murariui* Ortiz & Lalana, 1997 | 1.1 | 0.0 | 0.0 | 0.0 | 0.0 | 0.5 |
